# How Will We Dine? Prospective Shifts in International Haute Cuisine and Innovation beyond Kitchen and Plate

**DOI:** 10.3390/foods9101369

**Published:** 2020-09-26

**Authors:** Nele Schwark, Victor Tiberius, Manuela Fabro

**Affiliations:** 1 Faculty of Economics and Social Sciences, University of Potsdam, 14482 Potsdam, Germany; nschwark@uni-potsdam.de; 2 Institute of History and Philosophy of Science, Technology, and Literature, Technical University of Berlin, 10623 Berlin, Germany; manuela.fabro@yahoo.de

**Keywords:** Delphi method, fine dining, haute cuisine, high gastronomy, innovation, Michelin star, nouvelle cuisine, restaurants

## Abstract

Haute cuisine, the cooking style for fine dining at gourmet restaurants, has changed over the last decades and can be expected to evolve in the upcoming years. To engage in foresight, the purpose of this study is to identify a plausible future trend scenario for the haute cuisine sector within the next five to ten years, based on today’s chefs’ views. To achieve this goal, an international, two-stage Delphi study was conducted. The derived scenario suggests that the coronavirus disease 2019 (COVID-19) pandemic will lead to significant restaurant bankruptcies and will raise creativity and innovation among the remaining ones. It is expected that haute cuisine tourism will grow and that menu prices will differ for customer segments. More haute cuisine restaurants will open in Asia and America. Local food will remain a major trend and will be complemented by insect as well as plant-based proteins and sophisticated nonalcoholic food pairings. Restaurant design and the use of scents will become more relevant. Also, private dining and fine dining at home will become more important. The scenario also includes negative projections. These findings can serve as a research agenda for future research in haute cuisine, including the extension of the innovation lens towards the restaurant and the business model. Practical implications include the necessity for haute cuisine restaurants to innovate to cope with increasing competition in several regions. Customers should be seen as co-creators of the value of haute cuisine.

## 1. Introduction

Haute cuisine—the cooking style for fine dining at gourmet restaurants—addresses a discerning consumer segment used to extraordinary ingredients, complex and elaborate preparation, careful presentation, and outstanding service [1]. However, due to high consumer expectations and strong competition in the sector [2], haute cuisine requires pronounced creativity and innovation [3,4,5,6,7,8,9,10,11,12,13,14,15,16]. This innovation is not limited to food only but might also include the renewal of the restaurant business model [17]. Innovation and therefore change in haute cuisine are mainly driven by the chefs and restaurant employees [18]. Chefs such as Ferran Adrià are even considered institutional entrepreneurs who change the whole haute cuisine sector [19,20]. Consumers usually do not play an important role in the innovation process [16,18]. However, innovations have to be accepted by the consumers [21,22]. A limiting factor is the diverse diets or food movements, such as fruitarian, gluten-free, halal, kosher, local, low-carb(ohydrate), low-fat, organic, ovo, paleo, permaculture, raw, slow, vegan, or vegetarian food, to which consumers are associated [23,24].

As a consequence of the high innovation rate, haute cuisine is ever-changing. Trends, such as molecular cuisine, come as fast as they go [21,25,26,27,28]. This paper does not focus on understanding past changes of the industry but explores how it might further develop in the future. The research goal is to identify a plausible future trend scenario for the haute cuisine sector in the next five to ten years. More specifically, the paper addresses the consequences of the coronavirus disease 2019 (COVID-19) pandemic for haute cuisine restaurants, changes in customer segments, regional development, changes regarding food and beverages, and the role of enhanced dining experiences. To archive the research goal, an international two-stage Delphi study was conducted.

Such strategic foresight is highly relevant because it aims “to support decision making, improve long-term planning, enable early warning, improve the innovation process, and improve the speed in reacting to environmental change” [29]. Specifically, in haute cuisine, the identification of a trend scenario can help to anticipate upcoming changes in the industry to be able to adjust to them, either following or deliberately bucking these trends. Berghaus et al. stress that, especially in the luxury industry, this future perspective is necessary in order not to miss forthcoming market changes and to therefore risk restaurants’ survival [30]. To the researchers’ knowledge, such a study does not exist for the haute cuisine sector yet. Foresight, in the sense of exploring the future of haute cuisine, can create an advantage over competing chefs who lack this view [31,32].

The paper is structured as follows: In the next section, haute cuisine is addressed more closely. Then, the Delphi method and the conduct of this study, including the formulation and justification of the 31 projections, are explained. The projections represent statements that might become relevant to the future of haute cuisine within the next five to ten years and are assessed by experts regarding their occurrence. Then, the results are presented as descriptive statistics and the derived future scenario, from today’s point of view. The discussion of the findings and the conclusion complete the paper.

## 2. Background: Haute Cuisine

Haute cuisine is a French style of cooking and is regarded worldwide as a culinary reference for fine dining. The origins of haute cuisine trace back to the 17th century. With the publication of François Pierre de La Varenne’s culinary set of rules “Le Cuisinier François” in 1651, innovative techniques and methods for the preparation of meat, the use of roux, and the production of broth became a subject of discussion. The refinement and modernization of haute cuisine is still the goal of many cookbook authors. For example, in his cookbook “Les Dons de Comus”, François Marin calls for more elegant preparation and careful use of spices [33].

With the beginning of the French Revolution, the first major steps in the development of haute cuisine started to emerge. Many nobles were expropriated, and chefs lost their jobs [34]. As a result, most chefs started working in hotels, restaurants, and clubs. Some left France for the UK, Italy, Germany, and the USA Public restaurants emerged that now serve the general public. The complex dishes of haute cuisine became goods that are offered in the commercial milieus of European cuisine [33]. Master chefs such as Marie-Antoine Caréme and Auguste Escoffier developed classic recipes and started to restructure kitchen work. Their cookbooks are still considered the ultimate culinary standard. The subsequent generation of chefs such as Paul Bocuse, Pierre Troisgros, and Michel Guérard henceforth coined the term “Nouvelle Cuisine” [33].

The success of an haute cuisine restaurant is strongly influenced by the ratings of the most important gastronomic guides. One of the most important and well-known culinary restaurant guides is the Guide Michelin, which awards one to three stars [1]. The Guide Michelin is often used as a global benchmark for chef and restaurant ratings. The Michelin group published the first Guide Michelin in 1900. Drivers were given practical information on where to find good repair and service points for their car and where to get good quality accommodation and food. Since then, the Guide Michelin has developed into a prestigious ranking of fine dining and cuisine and has an international influence on haute cuisine [35,36]. One Michelin star stands for restaurants that are worth a stop, two stars are worth a detour, and three stars justify a trip just for dining [37]. The Gault-Millau is also considered a respected restaurant review with a focus on Europe and awards 1 to 20 points, whereas usually only restaurants with at least 10 points are listed. Whereas the Guide Michelin also reflects the service and atmosphere of the restaurant, the Gault Millau only focuses on food quality. Even though fine dining is an experience of all senses, food quality has the highest impact on customer satisfaction [38,39] and is therefore important for customer loyalty [40]. In the authors’ perception, the Guide Michelin cares more about the exquisiteness of the dishes and the Gault Millau focuses on innovativeness and uniqueness.

As another approach, The World’s 50 Best Restaurants are ranked based on a poll of international chefs, restaurateurs, gourmands, and restaurant critics. Despite its name, the list was extended to 120 restaurants. Most of 2019′s top 50 restaurants were located in Spain (7), the USA (6), and France (5). Two top 50 restaurants can be found each in China, Denmark, Italy, Japan, Mexico, Peru, Russia, Thailand, and the UK. Argentina, Austria, Belgium, Brazil, Chile, Colombia, Germany, The Netherlands, Portugal, Singapore, Slovenia, South Africa, Sweden, and Switzerland account for one top 50 restaurant each.

Another interesting ranking is La Liste, which calculates scores based on more than 600 international restaurant guides, consumer rating sites such as TripAdvisor and Yelp, and restaurant reviews from newspapers and magazines. According to their trustworthiness, different weights are ascribed to these sources. Due to this methodology, the list can be regarded as relatively objective. The 2020 list comprises more than 1000 restaurants which have ratings of at least 75%. The highest score of 99.50% was achieved in 2020 by Guy Savoy (Paris, France), Le Bernardin (New York, NY, USA), Ryugin (Chiyoda-ku, Japan), and Sugalabo (Minato-ku, Japan). Japan, China, France, the USA, and Spain account for most of the top restaurants, according to La Liste.

## 3. Method

### 3.1. The Delphi Technique

In order to formulate a plausible future trend scenario for haute cuisine in the next five to ten years, a two-stage Delphi study was carried out. The popular method was used 175 times in scholarly articles in business and management between 1975 and 2017 [41]. In contrast to scenario analyses [42,43,44,45], which generate several future scenarios, Delphi studies aim to identify the one most likely scenario, from today’s experts’ perspectives [46,47,48,49,50,51,52,53].

The Delphi method is suitable for forecasting social developments [54]. In contrast to developments which follow natural laws such as the weather, social changes occur due to human intentions, social interactions, and coincidences [55]. To forecast them, therefore, no clear causalities can be used. Rather, the Delphi method surveys experts about their subjective knowledge- and experience-based opinions over at least two rounds, using a standardized questionnaire and giving structured feedback about the results from the prior round to enhance the group consent [52,53,54,56,57].

The method builds on the idea that group assessments about projections (statements about the future) are more accurate than individual assessments and that experts can give more accurate assessments than laypersons [58,59]. As cognitive biases are reduced during the Delphi process [58,60,61,62], Delphi studies are attributed a high forecast accuracy [63].

### 3.2. Formulation of Projections

Since the future of haute cuisine has not yet been the subject of a Delphi study or other prospective studies, a broad, explorative rather than a narrow and deep scenario approach was chosen for this study [52,64]. A broad Delphi approach covers many specific aspects but leaves out details. Later, Delphi studies can choose narrower areas and scenarios and can ask more detailed questions. The choice between the two approaches is necessary because the number of projections must be appropriate. That way, a high number of responses can be generated and the dropout rate can be kept as low as possible.

The projections of Delphi studies are formulated on the basis of plausible conclusions from current developments. However, implausible projections can also be included, as the same conclusions can be drawn from the assumption of a plausible projection or by rejecting an implausible projection [52]. Thirty-one projections for five selected topics were formulated. As projections for Delphi studies are kept short and unambiguous [65,66], particular attention was paid to the explicit wording. Each projection contains only one statement. Otherwise, conflict could arise where some respondents agree with one statement but reject the other and the entire projection is subsequently rejected [52].

#### 3.2.1. Current Pandemic

Possible long-term economic impacts from the COVID-19 pandemic on restaurants need to be taken into consideration. Gössling et al. assume that restaurants will have problems recovering after the pandemic, as they usually have limited liquidity and low profit margins [67]. P1: In the next five to ten years, crises like the corona virus pandemic will push a significant part of haute cuisine restaurants out of the market.

However, times of crisis can also increase creativity and therefore innovation [68,69]. Turbulent times might even trigger radical innovations [70,71] that lead to market disruptions [72,73]. Creativity and innovation are considered important antecedents of competitive advantages, especially for restaurants [2,12,36]. Therefore, the crisis could even increase some restaurants’ performance [74]. P2: In the next five to ten years, crises like the corona virus pandemic will significantly increase creativity and innovation in haute cuisine restaurants.

#### 3.2.2. Customers

As many luxury segments, such as in fashion [75] or tourism [76], have been growing, it can be expected that haute cuisine will also attract more customers in the future. P3: In the next five to ten years, the number of customers visiting haute cuisine restaurants will increase significantly.

The Guide Michelin’s definition of a three-star restaurant is that it is worth a trip [37]. Especially, The 50 World’s Best Restaurants are mainly visited by international rather than local guests. Culinary travelers’ main reason for tourism is fine dining [77]. Waiting lists of over a year at the most successful restaurants show that haute cuisine tourism might grow in the future. P4: Over the next five to ten years, haute cuisine tourism will grow.

Segmentation of the market and targeting specific consumer groups is a key concept in marketing [78]. Apart from the usual demographic measures to distinguish segments, restaurants can segment the market by considering the diets and food movements to which consumers ascribe. For example, three-star chef Dominique Crenn, in her restaurants in San Francisco, has only served vegetarian dishes since 2019 [79]. P5: Within the next five to ten years, a large part of haute cuisine restaurants will only address certain target groups or nutritional styles, such as fruitarian, gluten-free, halal, kosher, low-carbohydrate, low-fat, vegan, ovo, vegetarian, paleo, raw food, etc.

Haute cuisine restaurants have always predominantly addressed consumers with a higher income level. Due to the emergence of the even narrower customer segment for ultra-luxury [80], it could be expected that there will be restaurants that only specialize in a particularly high wealth level and therefore very high passive income, such as Ultra High Net Worth Individuals (UHNWI), who have a net worth of at least US$ 30 million. The Knight Frank Wealth Report 2020 estimates that 513,244 individuals can be categorized as UHNWIs globally at the end of 2019 [81]. Additionally, the opposite could also be expected, leading to a broader price range in haute cuisine. P6: In the next five to ten years, the range of menu prices will expand for different income levels. P7: In the next five to ten years, there will be restaurants that focus on UHNWI (Ultra High Net Worth Individuals) as a target group and will have corresponding prices.

#### 3.2.3. Regional Developments

For a long time, France was the central focus of haute cuisine. This is where the upscale dining culture emerged and set a worldwide standard for subsequent upscale cuisine. However, two- and three-star restaurants can now be found in many countries around the globe [37]. Currently, there are even more three-star restaurants in Tokyo than in Paris [37]. For this reason, it is worth considering future possible developments within different regions. P8: Over the next five to ten years, the number of haute cuisine restaurants in Africa will increase significantly, increase slightly, not change, or decrease significantly. P9: Over the next five to ten years, the number of haute cuisine restaurants in Central Asia will increase significantly, increase slightly, not change, or decrease significantly. P10: Over the next five to ten years, the number of haute cuisine restaurants in East Asia will increase significantly, increase slightly, not change, or decrease significantly. P11: Over the next five to ten years, the number of haute cuisine restaurants in Southeast Asia will increase significantly, increase slightly, not change, or decrease significantly. P12: Over the next five to ten years, the number of haute cuisine restaurants in Western Asia will increase significantly, increase slightly, not change, or decrease significantly. P13: Over the next five to ten years, the number of haute cuisine restaurants in Europe will increase significantly, increase slightly, not change, or decrease significantly. P14: Over the next five to ten years, the number of haute cuisine restaurants in Central and South America will increase significantly, increase slightly, not change, or decrease significantly. P15: Over the next five to ten years, the number of haute cuisine restaurants in North America will increase significantly, increase slightly, not change, or decrease significantly. P16: Over the next five to ten years, the number of haute cuisine restaurants in Oceania will increase, increase slightly, not change, or decrease significantly.

#### 3.2.4. Food and Beverages

More and more consumers want to know where their food comes from and how it is produced, and transparency in the handling of food is becoming increasingly important. Previous studies have shown that individuals prefer locally grown foods due to their freshness, familiar taste, and sustainability, as long-distance transportation is not necessary. This trend also prevails in haute cuisine. However, conceptually, local food does not relate to exclusivity. Therefore, there is a chance that this trend might end. P17: In the next five to ten years, the trend towards local food will decline and ingredients from distant regions will become more important.

With a few exceptions, the molecular cuisine as an avant-garde movement is largely out of fashion. Phil Howard, a world-renowned chef, told “CLH News”: “However, I think as things go in cycles and you look at techniques such as molecular gastronomy then the pendulums swings pretty swiftly back to where it once was, and whilst we have learned some great things in that journey, people have come back to caring and buying locally, buying quality ingredients and treating them quite simply” [82]. However, as naturalness is also a trend that came and went throughout history, here is a chance for its revival. P18: Molecular cuisine or similar approaches will return within the next five to ten years.

Current discussions on climate change illustrate the urgency to develop sustainable meat alternatives, such as cultured or in vitro meat, and to bring them to the market [53,83]. However, consumer acceptance is very heterogeneous [84]. On the one hand, haute cuisine could take up this meat alternative due to its pioneering role. On the other hand, this would contradict the current trend of naturalness. P19: Cultivated or in vitro meat will be served within the next five to ten years.

Additionally, products based on insect proteins represent a sustainable alternative to meat. They are rich in proteins and provide unsaturated fatty acids and micronutrients [85]. The biggest challenge will be consumer acceptance [86,87,88]. P20: Insect-based proteins will be served within the next five to ten years.

Apart from the abovementioned alternatives, also consumer demand for plant-based proteins is rising [89,90]. This trend might continue and have an influence on haute cuisine. P21: Vegetable proteins will be served within the next five to ten years.

Haute cuisine menus are traditionally paired with fine wines [91,92,93]. However, not all consumers prefer alcoholic beverages [94]. For them, apart from water, some haute cuisine restaurants started to offer nonalcoholic dish-wise pairings based on juices and herbs, which can be considered creative and complex. This could become a new standard for haute cuisine restaurants. P22: In the next five to ten years, the production of nonalcoholic beverages (as a food pairing) will be as complex and sophisticated as the production of food.

#### 3.2.5. Experiences

Visiting an haute cuisine restaurant is a culinary experience which has little in common with dining at a normal restaurant. While some restaurants focus on the food alone, others follow a more holistic approach [95] as they address more than just the sense of taste. In addition, they might address further senses such as sight. Multi-sensory experiences play an increasingly important role in everyday life [51] and might also become more relevant in haute cuisine. P23: Over the next five to ten years, different designs and styles of restaurants will play a more important role and become more different from each other.

Apart from taste and sight, smell and hearing could also be addressed. Both scent and background music have an impact on customer behavior and satisfaction [96]. Consumers associate different bars and dishes with certain scents and musical accompaniments [97]. P24: In addition to taste, scent will play a more important role in the next five to ten years. P25: In the next five to ten years, music and sounds will play a more important role in addition to taste.

Rather than addressing senses individually, their complex interplay has to be noted. A holistic approach could be the inclusion of a concerted entertainment concept. For example, Rasmus Munk’s restaurant Alchemist in Copenhagen involves theatre and art as parts of a holistic dining experience. Whereas this is a rare example, more restaurants might follow. P26: Over the next five to ten years, a significant number of restaurants will offer haute cuisine in combination with entertainment.

Another way to expand customers’ experience during their stay at a restaurant would be their active involvement, as suggested in other industries by a prosumer approach [98]. The involvement of customers increases their opinion about the meal [99]. It might even enhance the restaurant’s creativity and innovation [100] and therefore be the foundation of an open innovation strategy [101,102,103,104,105,106,107,108,109,110] and a business model based on value co-creation [111,112,113]. P27: Over the next five to ten years, a significant proportion of haute cuisine restaurants will integrate customers as prosumers, meaning they participate in the preparation of their own dishes.

The sharing economy made the idea of sharing not only a major economic but also cultural trend [114,115,116,117,118,119,120,121]. Therefore, also food sharing within the restaurant is also becoming increasingly popular [122]. Plates with prepared food are put on the table to encourage customers to help themselves. The Swiss chef Andreas Caminada is also following this concept with his two-star restaurants Igniv in St. Moritz and Bad Ragaz. That way, customers do not have to choose between menus and chefs have the opportunity to showcase more facets of their culinary art [37]. However, it is unlikely that this trend will also be applicable to guests who do not know each other as food sharing is attributed to intimacy [123]. P28: In the next five to ten years, food sharing between customers (who do not know each other) will become more important.

In addition to such experiences within the restaurant, fine dining restaurants could also extend their service to before and after the stay, by offering a limousine service. Some luxury hotels already provide their guests with a limousine service that takes them to gastronomic highlights [124]. P29: Over the next five to ten years, a significant proportion of haute cuisine restaurants will offer first-class limousine services that will pick up customers and bring them home.

Another trend is “private dining”, both in separate rooms within the restaurant and at home. P30: In the next five to ten years, private dining in separate rooms of the restaurant will become more important. P31: Over the next five to ten years, a significant proportion of the (former) haute cuisine chefs will cook at customer’s homes and not at restaurants.

### 3.3. Expert Panel

Delphi studies are based on purposive sampling [125]. Only experts with expertise in this field should be selected for these samples [62,126,127,128]. The experts in this study were selected on the basis of their entry on the official Guide Michelins website. All restaurants with one, two, or three stars were contacted by email, as fast as this contact was offered, and the website was offered in English. The focus on the restaurant staff as the expert group is appropriate as they are the main drivers of innovation in haute cuisine, rather than consumers [6,18] or critics.

As a result, a total of 2427 potential respondents were contacted. Of them, 13 could not be reached and 19 said they did not want to be contacted again. Of the remaining 2395, 72 experts participated in the first round with 60 responses completed, and in the second round, 23 experts participated with 22 responses completed. Detailed demographic data for the panel are shown in Table 1. The very low participation rate of women is striking but reflects the reality in haute cuisine.

As the clear majority of participants stem from European countries, the study has a bias towards Europe. However, the Asian as well as North and South American respondents partly reduce this cultural bias.

### 3.4. Data Collection

The experts were asked to evaluate the 31 projections in the five thematic sections outlined above. The questionnaire was in English. The extent of (dis)agreement with the individual statements was to be expressed on a four-point-Likert-scale (“fully agree”, “agree”, “disagree”, and “absolutely disagree”). For Section 3, the Likert scale was adjusted (“increases strongly”, “increases slightly”, “does not change significantly”, and “decreases”). The straight scale in both cases was used to avoid a trend towards the center. In addition to the ratings above, experts were asked to provide demographic information such as gender, age, location, and status within the restaurant.

After performing a pretest to minimize risk of misinterpretation or technical problems with the survey tool, the first round of the survey was conducted from 23 April to 7 May, 2020. The second round started on 14 May and ended on 11 June 2020. For every projection, the results from the first round were presented visually. The average time to complete the survey was 6 min and 11 s in the first round and 6 min 48 s in the second round. The slightly longer duration for the second round can be explained by the need to grasp the visually presented results of the first round for each projection. The study was carried out anonymously, i.e., the respondents were not asked to provide their names when submitting the questionnaire, because anonymity is considered a key feature of Delphi studies [54].

## 4. Results

### 4.1. Descriptive Statistics

To calculate the group consensus, the answer options received the following numerical values: 1: “do not agree at all”, 2: “do not agree”, 3: “agree”, and 4: “fully agree” or, in Section 3, 1: “decreases”, 2: “does not change significantly”, 3: “increases slightly”, and 4: “increases strongly”.

To avoid a statistical bias, the median was used as the preferred statistical average for Delphi studies because it is more robust to outliers than the mean [129]. To interpret the aggregate responses, scattering is measured by the interquartile range (IQR) as the difference between the upper quartile (x_0.75_) and the lower quartile (x_0.25_). A small IQR stands for a high level of consensus, whereas a high IQR stands for a high level of dissent. The aim is to reduce scattering around the median (x_0.5_) in the course of Delphi iterations.

None of the projections received full approval (x_0.5_ = 4) or full rejection (x_0.5_ = 1). The experts agreed on projections 1–4, 6, 7, 20–24, 30, and 31 (x_0.5_ = 3). They did not agree with projections 3, 5, 17–19, and 26–29 (x_0.5_ = 2). Projections 9–12, 14, and 15 were rated as “increases slightly” (x_0.5_ = 3), and projections 8 and 13 were rated as “does not change significantly” (x_0.5_ = 2). The median of projections 16 and 25 was ambiguous or neutral (x_0.5_ = 2.5).

In the second round, the median shifted from 2 to 3 for projections 4 and 20, indicating that the previously rejected projections were now approved. The same shift in median from 2 to 3 also applied to projections 9, 14, and 15, indicating a slight growth rather than no significant change in the corresponding regions. No changes occurred for the other projections.

The overall IQR of the projections was 1.05 in the first and 0.61 in the second round, which can be considered a fairly high group consensus. The average IQR difference was −0.44, which shows that the desired IQR reduction occurred. As all IQRs were 1 or less, the iteration could be stopped after the second round. The scattering of opinions decreased for 16 projections. In particular, for projections 5, 12, and 17, the IQR was reduced by 0.25. The IQR decreased by 1.00 for projections 6, 7, 10, 11, 13, 15, 19, 23, 24, 27, and 28, and by 1.25 for projections 1 and 2. Scattering only increased for projection 21, indicating a slight increase in insecurity. Detailed results of the Delphi study for both rounds are shown in Table 2.

### 4.2. Scenario

Experts believe that pandemics like the current COVID-19 pandemic will have an impact on haute cuisine over the next five to ten years. They will push restaurants out of the market but, at the same time, promote creativity and innovation.

According to the experts, the number of people visiting haute cuisine restaurants will not increase significantly. The experts also believe that specialization of individual restaurants in certain diets or food movements, such as fruitarian or vegan diets, is unlikely. Experts see more potential in culinary tourism related to haute cuisine. Here, they see growth and growing importance for the next five to ten years. Furthermore, it is assumed that menus in different price segments will address different income levels. Some restaurants might even focus exclusively on UHNWI as a target group.

The number of restaurants worldwide will increase, with Central Asia, East Asia, Southeast Asia, West Asia, and South and North America seeing a slight increase in haute cuisine restaurants. In contrast, experts agree that neither Africa nor Europe can expect a significant increase. The experts expect that the trend towards local food will continue and that ingredients from distant regions will not play a more important role. Past trends such as molecular cuisine will not experience a renewed upswing. As far as alternative protein sources are concerned, it is predicted that vegetable and insect-based proteins will be served rather than in vitro meat. In addition, it is expected that the creation of nonalcoholic beverages as an alternative food pairing will be just as complex and sophisticated as wine.

The haute cuisine restaurants of the future will increasingly differentiate themselves and develop different designs and styles. In addition to taste experience, smells and possibly background music will become more important. Even if scents and more private environments will play an important role, the majority of restaurants will not combine restaurant visits with some kind of entertainment. However, sharing food among customers who did not know each other before visiting the restaurant will not play a significant role. Customers also will not participate in the preparation process. Offering a limousine service that picks up customers and brings them back home will also not become a standard service. However, private dining will become more important. There will even be a significant proportion of (former) haute cuisine chefs who cook at customers’ homes instead of at a restaurant.

## 5. Discussion

### 5.1. Rejected Projections and Shifts

All projections were formulated in a way that their approval could have been expected. Whereas 18 projections were accepted, 11 were rejected and 2 were rated neutrally. In the following, therefore, the main focus is on the rejected and neutrally rated projections.

In the pandemic section, experts agreed to both upcoming bankruptcies and the fostering effect on creativity and innovation. However, these assessments have to be viewed with caution as assessing these projections right at the beginning of curfews and lockdowns is generally difficult since respondents are not objective observers but are directly affected in regard to their economic existence by state measures to cope with the pandemic. However, as the two projections address two contrasting outcomes, namely a negative one (bankruptcies) and a positive one (creativity and innovation), and both were agreed to, no pessimistic bias appears in the assessments.

In the customer section, the panel did not expect a growing number of haute cuisine customers. This assessment somewhat contradicts the expectation that the number or restaurants will grow in most countries. This would mean that more restaurants serve a steady or even decreasing number of customers, at least in most regions. However, the projection regarding target group growth is not regionally differentiated (as Section 3 is). It can be assumed that the experts had their home region in mind when assessing this projection. As most experts are located in Europe, the assessment is coherent with projection 12, which expects a decline rather than a rise in the number of restaurants. The second projection in this section that was rejected is projection 5, which stated that restaurants will specialize in specific diets or food movements. This assessment is plausible considering that haute cuisine already serves a niche market. Another sub-segmentation of this small segment would probably not be economically reasonable. Only for vegetarians, this trend is feasible and already being practiced. Interestingly, from the first to the second rounds, a median shift from disagreement to agreement occurred for projection 4, addressing a possible growth of haute cuisine tourism. Therefore, while the overall number of customers is not expected to increase, their mobility is. According to the Guide Michelin definition of their ratings, this specifically concerns three- and two-star restaurants. Certainly, especially the restaurants listed as The World’s Best 50 Restaurants will be on haute cuisine travelers’ bucket lists.

In the regional section, apart from the aforementioned rejection of growth projection for Europe, also no growth is expected for Africa, which is not surprising as the haute cuisine culture is very small. However, it could also be argued that its growth potential is higher just because it is currently very small. However, when rating the projection, the panel probably interpreted that growth leading to a level that can be compared to other regions worldwide, which is not likely in the short term. For Oceania, the experts have no clear expectations. The reason for this is probably that the region is not internationally renowned for haute cuisine. In general, the ratings have to be viewed with certain caution because some respondents might have mentally replaced the continents with specific countries and rated the projections for them. Depending on personal connections (e.g., vacations), these assessments could differ from respondent to respondent. In this section, the panel changed their growth expectations from the first to the second rounds for several regions, namely Central Asia, Central and South America, and North America, which formerly were not seen as having growth potentials but then were ascribed to future growth. Therefore, six out of nine regions were formerly seen as stagnating or shrinking, whereas in the second round, seven regions were seen as more positive than before. A reason for this might be that the COVID-19-related pessimism was stronger during the first survey round compared to the second one. For the sake of wording, however, it should be noted that the projections in this section relate to significant growth. By rejecting this projection, no statement is made about a possible (slight) growth.

In the foods and beverages section, the panel expects the trend towards local food to persist. The exclusive character of ingredients from far-distanced places is probably beaten by the sustainability trend because of two reasons: First, sustainability is an omnipresent societal imperative that receives utmost attention across all areas of social life, also in the luxury industry [130,131,132,133,134,135] and, within in, in haute cuisine [136]. Second, ingredients from exotic countries do not have the exclusive appeal they used to have as most customers are aware that, in the era of globalization and international supply chains [137,138,139,140], even many fruits and vegetables in common supermarkets have been transported over far distances and are usually still not more expensive than local ones. The panel also sees no upcoming return of molecular cuisine. Whereas this avant-garde, scientific cuisine received strong attention due to its complete otherness, it never became as popular on real plates as in the media. For the majority of consumers, molecular cuisine’s artificiality was too extraordinary rather than interesting. Whereas molecular cuisine is the logical evolution of haute cuisine in regard to its innovativeness compared to “common” food, consumers might not want to go this further step (yet). Additionally, among the little number of chefs who could be ascribed to molecular cuisine, some, such as Juan Amador, even reject this ascription. Lastly, in this section, the panel does not see the serving of in vitro meat coming up in the near future. On the one hand, whereas cultured meat has been subject to intense scientific research for quite a time now [53,141,142,143,144,145,146], its practical large-scale application for the mass market is not yet in sight. On the other hand, it contradicts the demand for naturalness, as it is conceptually not far from the perceived artificiality of the aforementioned molecular cuisine. The only median shift and therefore change of group opinion occurred for projection 20 regarding serving insect-based proteins, which was accepted in the second round. It is hard to tell why this shift occurred, but as insects as food have been increasingly discussed in recent years [86,147,148], this assessment is likely to turn out true. Interestingly, projection 21 focusing on plant-based proteins is the only one that received a slight increase in IQR, suggesting a higher level of insecurity in the second round. A reason for this might be that it might not have been clear if the projection’s focus is on vegetarian food or on proteins. Haute cuisine is more concerned with the taste of food rather than its nutritional value, i.e., haute cuisine restaurants do not serve meat because it is, for example, low-carb, but because of its taste. Therefore, the question stressing proteins might have been somewhat confusing.

In the experiences section, the panel acknowledges the arrival of several augmented experiences but are skeptical when the extensions clearly exceed today’s standards. For example, while the experts agree to an increased relevance of design and scents, they are undecided regarding music and sound and reject the idea of even more entertainment in haute cuisine restaurants. As stated above, several avant-garde restaurants already see the inclusion of visual and auditory senses as parts of their holistic dining experience. In contrast, the majority of experts expect that food will still remain the main aspect, probably because other senses are rather considered to distract from this original experience. In coherence with this rather conservative expectation, the panel also does not see that the customer will interact with chefs in food preparation or with unknown guests by sharing food. Therefore, dining at an haute cuisine restaurant is expected to remain an activity centered on food that is solely experienced jointly with the guest’s company. Finally, the experts do not see an extension of the restaurants’ service towards a transfer from home to the restaurant and back. Again, this expresses the panel’s expectation that haute cuisine restaurants will mainly stick to their core competencies. The only striking shift in this section concerned projection 25, stating an increasing relevance of entertainment. The panel first agreed to it and then turned to undecided. The experts possibly did not see a large difference between this projection and projection 26 on entertainment, which was, as mentioned above, rejected.

Overall, the panel confirms trends that are already visible but is skeptical about possible changes which deviate too much from the current standards. Over the next five to ten years, haute cuisine will, according to experts, continue to follow the maxim: “Let the cobbler stick to his last,” and the evolution is expected to be gentle rather that disruptive. Considering the fact that haute cuisine has its roots not only in luxury but also in innovation, the conservative expectations for its future development are somewhat counterintuitive. However, innovation mainly occurs in the kitchen and on the plate, and even there, some frontiers are hard to overcome, as could be seen with molecular cuisine. The further context beyond kitchen and plate is rather stable. Haute cuisine, as many other industries, shows a high degree of institutional isomorphism, i.e., restaurants are quite similar in structural and processual regards [149]. In addition, their business model is practically identical. Only a few try to break out of the institutional norms, and even fewer try to innovate their business model. To be further distinguished in the highly competitive market, the vanguard of haute cuisine might question many established characteristics of haute cuisine in particular but possibly even of dining in general. Several of the projections addressed a few possibilities for this. However, many more exist.

### 5.2. Limitations

As with all research, this study also has some limitations. First, it must be emphasized again that cultural (as opposed to many natural) phenomena in general cannot be predicted with absolute certainty [150]. Delphi studies are a systematic method that, with the help of expert opinions, generates the most likely future scenario. Future research could also use other methods such as prediction markets [151,152]. Additionally, Delphi scenario only describes a probable future but does not theoretically explain how it emerges [153,154].

Second, Delphi studies are confronted with some general critique. For example, Goodman and Sackman criticize the fact that the exposure of interim results violates the scientific requirements of an independent judgment [155,156]. However, the goal of a Delphi study is the convergence of opinions expressed and the reduction of scattering in order to reach consensus [52]. Another issue of Delphi studies is the desirability bias, which means that the experts might rate the projections according to their subjective hopes and wishes rather than the objective expected occurrence [60,62]. This problem diminishes, like in this study, with a higher number of experts, who are unlikely to have the same hopes and wishes for the future. Besides, experts might also rate according to their fears. Delphi studies are also criticized because the iterative rounds might not lead to reduced scatterings due to a better assessment of the projections but due to group pressure [62,129,157,158]. A closer look at the differences between the two rounds, however, shows median shifts, which suggests that the experts changed their opinions despite prior group consensus.

Third, a single Delphi study cannot cover a subject area both comprehensively and in detail at the same time [52]. In order to avoid low response and repetition rates, an attempt was made to keep the survey as short as possible. For this reason, it was not possible to investigate all relevant topics that would have been possible for a more embracing forecast of the future of haute cuisine. Against this background, this Delphi study provides a first overview of the developments to be expected for the next five to ten years, divided into the addressed five thematic sections. Further future developments within haute cuisine restaurants and approaches to creativity and innovation have not been taken into account but may lead to interesting results in future studies. Future research could examine one or more of the five thematic sections of this study in greater detail and could gain deeper insights or even address new subject areas.

Fourth, the response and repetition rates have to be viewed critically. The absolute numbers of respondents can be considered satisfactory, as they are usually small [53,127,159]. The minimum should be seven participants [160], whereas most Delphi studies have a panel size of 15 to 35 [126]. However, the relative numbers have to be considered low, with a response rate of 3.01%/2.51% (gross/net) and a repetition rate of 36.7%. The main reason for this might be that the surveys took place during the curfews and lockdowns that many countries imposed in response to the COVID-19 pandemic, which challenged many small businesses in several ways [67,161] and therefore also restaurants. While it may be argued that the chefs and other restaurant staff had enough time to complete the assessments of the projections, their motivation to support research could be expected to be somewhat low as the lockdowns threatened restaurants’ economic survival, as also addressed in opening projection P1. A scenario developed based on a Delphi study before or some months after the crisis might look different. Additionally, the survey was conducted in English, which might have caused language problems for participants in many countries. Another reason could be that the number of propositions was too high and demotivated the respondents to participate (see the third limitation). An indicator for this could be that 12 respondents did not finish the survey.

Fifth, the expert panel only comprised haute cuisine staff but no other stakeholder groups. Future research could also include other potential respondents such as frequent haute cuisine customers and restaurant critics.

Sixth, the distribution of experts across the different regions was very uneven. The reason for this might have been the aforementioned language barriers. It is especially unfortunate that no respondents from Japan participated in the study as Japan is a leading hot spot for haute cuisine. Also, the lack of responses from Africa is regrettable. Therefore, the assessment of P8 has to be viewed with certain skepticism. Therefore, although the study examines international developments and addresses important aspects, a broader panel of experts which would be better distributed among the regions might have resulted in other future scenarios. It would have been interesting to see if certain regions developed different common consensuses.

### 5.3. Implications

The findings have theoretical and practical implications. For scholars researching haute cuisine, the depicted scenario and, more specifically, the approved projections indicate themes which are worth further inquiry in the future. Therefore, this study can also serve as a research agenda. In addition, haute cuisine research has a strong focus on innovation. However, as discussed before, the innovation focus is mostly limited to food alone. Future researchers should broaden the perspective towards the restaurant structure and processes as well as business model innovation [17,162,163,164,165]. The still neglected integration of the customers into the innovation process seems to be imperative as they are an important driver to enhance the agility in the front end of innovation [166,167,168,169,170,171].

For chefs and other haute cuisine managers, the study suggests that the sector will slightly evolve. However, in regions with a growing number of restaurants, competition will increase and require intensified innovation efforts. As stated before, this should not only be limited to the kitchen and plate but also include the overall restaurant context and possibly even the business model. In that regard, a still underused potential is the involvement of customers in both meal innovation [6,18] and business model innovation [172]. However, even if customers are not involved in the innovation process, their role should be extended from that of pure consumers to those who co-create the value of haute cuisine [169,173,174].

## 6. Conclusions

This study provides a first holistic forecast regarding a range of themes in haute cuisine and can give insights into a plausible future over the next five to ten years. The derived scenario suggests that the COVID-19 pandemic will drive restaurants out of the market and encourage creativity and innovation among the remaining ones. It is not expected that haute cuisine will attract more customers in the future but that haute cuisine tourism will grow and that different price ranges will address different customer segments. More haute cuisine restaurants will likely open in Asia and America but less likely open in Africa and Europe. The trend towards local ingredients is intact, and fine dining will probably use insect- and plant-based proteins and offer sophisticated nonalcoholic beverages. In contrast, in vitro meat is less likely to arrive at haute cuisine restaurants, and a return of molecular cuisine is not expected. The enhancement of non-taste-related experiences is predicted to become more relevant, especially regarding restaurant design and the use of scents, but less likely of auditory sensations. Private dining and haute cuisine cooking at home will become more relevant.

In sum, the prospective changes in haute cuisine are expected to be somewhat small. Chefs will take up customers’ wants and needs, and innovation will focus on food but not concern the restaurant at large and not its business model. However, treating customers as the co-creators of the haute cuisine experience and involving them in innovation might offer interesting opportunities which are currently underused. For the vanguard of avant-garde restaurants, questioning the foundations of dining and disrupting the customers’ expectations will provide interesting outcomes.

## Figures and Tables

**Table 1:** Panel structure.

Demographic Details	First Round (N = 60)		Second Round (N = 22)	
**Gender**	***n***	**%**	***n***	**%**
Male	49	81.7	20	90.9
Female	6	10.0	2	9.1
No response	5	8.3	0	0.0
**Age**	***n***	**%**	***n***	**%**
Under 30	3	5.0	1	4.6
30–40 years	30	50.0	12	54.6
41–50 years	17	28.3	7	31.8
51–60 years	8	13.3	2	9.1
Older than 60 years	1	1.7	0	0.0
Not specified	1	1.7	0	0.0
**Status in restaurant**	***n***	**%**	***n***	**%**
Chef	23	38.3	13	59.1
Service management	1	1.7	0	0.0
Restaurant management	6	10.0	3	13.6
Restaurant management and chef	25	41.7	6	27.3
Not specified	5	8.3	0	0.0
**Continent**	***n***	**%**	***n***	**%**
Asia	5	8.3	2	9.1
Europe	45	75.0	18	81.8
North America	5	8.3	1	4.5
South America	2	3.3	1	4.5
Not specified	3	5.0	0	0

**Table 2:** Descriptive statistics.

Sections/Projections			First Round (N = 60)		Second Round (N = 2 2)					Differences			
			x_0.5_	IQR	x_0.5_			IQR		Δx_0.5_		ΔIQR	
**Section 1: Current pandemic**													
1. Many restaurants going bankrupt			3.00	1.25	3.00			0.00		0.00		−1.25	
2. Increasing creativity and innovation			3.00	2.00	3.00			0.75		0.00		−1.25	
**Section 2: Customers**													
3. Growing number of customers			2.00	1.00	2.00			1.00		0.00		0.00	
4. Growing haute cuisine tourism			2.00	1.00	3.00			1.00		1.00		0.00	
5. Targeting specific consumer segments			2.00	0.25	2.00			0.00		0.00		−0.25	
6. Differentiating menu prices			3.00	1.00	3.00			0.00		0.00		−1.00	
7. Some restaurants targeting only UHNWI			3.00	1.00	3.00			0.00		0.00		−1.00	
**Section 3: Regional growth**													
8. Growing number of restaurants in Africa			2.00	1.00	2.00			1.00		0.00		0.00	
9. Central Asia			2.00	1.00	3.00			1.00		1.00		0.00	
10. East Asia			3.00	1.00	3.00			0.00		0.00		−1.00	
11. South East Asia			3.00	1.00	3.00			0.00		0.00		−1.00	
12. Western Asia			3.00	1.00	3.00			0.75		0.00		−0.25	
13. Europe			2.00	2.00	2.00			1.00		0.00		−1.00	
14. Central and South America			2.00	1.00	3.00			1.00		1.00		0.00	
15. North America			2.00	2.00	3.00			1.00		1.00		−1.00	
16. Oceania			2.00	1.00	2.50			1.00		0.50		0.00	
**Section 4: Food and beverages**													
17. End of local food trend			2.00	1.00	2.00			0.75		0.00		−0.25	
18. Return of molecular cuisine			2.00	1.00	2.00			1.00		0.00		0.00	
19. Serving cultivated/in-vitro meat			2.00	2.00	2.00			1.00		0.00		−1.00	
20. Serving insect-based proteins			2.00	1.00	3.00			1.00		1.00		0.00	
21. Serving vegetable-based proteins			3.00	0.00	3.00			0.75		0.00		0.75	
22. Serving sophisticated nonalcoholic beverages			3.00	1.00	3.00			1.00		0.00		0.00	
**Section 5: Experiences**													
23. Design styles becoming more relevant			3.00	1.00	3.00			0.00		0.00		−1.00	
24. Increasing relevance of scents			3.00	1.00	3.00			0.00		0.00		−1.00	
25. Increasing relevance of music and sounds			3.00	1.00	2.50			1.00		−0.50		0.00	
26. Increasing relevance of entertainment			2.00	1.00	2.00			1.00		0.00		0.00	
27. Integrating consumers in dish preparation			2.00	1.00	2.00			0.00		0.00		−1.00	
28. Sharing food (between unfamiliar guests)			2.00	2.00	2.00			1.00		0.00		−1.00	
29. Offering first-class limousine service			2.00	1.00	2.00			1.00		0.00		0.00	
30. Increasing relevance of private dining			3.00	0.00	3.00			0.00		0.00		0.00	
31. Cooking at customers’ homes			3.00	0.00	3.00			0.00		0.00		0.00	
**Assessment (2nd round)**	agree/increase (a)	disagree/decrease (b)	neutral (c)		a	b	c						
**Assessment stability**	stable (d)		change (e)							d	e		
**Scattering (2nd) round)**	low (IQR ≤ 1) (f)		high (IQR > 1) (g)					f	g				
**Scattering reduction**	stable or decreased (ΔIQR ≤ 0) (h)		increased (ΔIQR > 0) (i)									h	i
**Notes**: x_0.5_ = median; IQR = interquartile range (x_0.75_–x_0.25_)													
